# 3. How comprehensive can we be in the economic assessment of vaccines?

**DOI:** 10.1080/20016689.2017.1336044

**Published:** 2017-08-31

**Authors:** Baudouin Standaert, Rino Rappuoli

**Affiliations:** a Health Economics, GSK, Wavre, Belgium; b Research & Development, Research Center, GSK, Siena, Italy

**Keywords:** Budget, economic evaluation, incremental cost-effectiveness ratio, societal perspective, vaccines, value assessment

## Abstract

In two previous papers we argued on current vaccines economic assessment not fully comprehensive when using the incremental cost-utility analysis normally applied for treatments. Many differences exist between vaccines and drug treatments making vaccines economic evaluation more cumbersome. Four challenges overwhelmingly present in vaccines assessment are less important for treatments: requirements for population, societal perspectives, budget impact evaluation, and time focused objectives (control or elimination). Based on this, economic analysis of vaccines may need to be presented to many different stakeholders with various evaluation preferences, in addition to the current stakeholders involved for drugs treatment assessment. Then, we may need a tool making the inventory of the different vaccines health economic assessment programmes more comprehensive. The cauliflower value toolbox has been developed with that aim, and its use is illustrated here with rotavirus vaccine. Given the broader perspectives for vaccine assessment, it provides better value and cost evaluations. Cost-benefit analysis may be the preferred economic assessment method when considering substitution from treatment to active medical prevention. Other economic evaluation methods can be selected (i.e. optimisation modelling, return on investment) when project prioritisation is the main focus considered and when stakeholders would like to influence the development of the healthcare programme.

## Introduction

Two previous papers identified various challenges for the total economic value positioning of a new vaccine entering the healthcare market [1,2]. Four factors have the greatest influence on its economic value performance: a new vaccine should preferentially be evaluated at the population level; it may have a broader societal impact than a treatment drug; budget analysis is a more critical factor for implementation of a new vaccine programme than the cost-effectiveness result; and the economic assessment of the short term could be very different from the long-term evaluation [3]. These four factors (population, society, budget, and timing) are linked through the performance of an analysis at a broader perspective than the one from healthcare only. As a consequence, the decision on whether to introduce a new vaccine may not remain a process in the hands of one entity. There could be many decision-makers involved, especially those not often considered in the current economic evaluations, such as Ministry of Finance, Ministry of Economics, and/or Ministry of Plan. Missing their viewpoint in the evaluation could have critical consequences for a new vaccine becoming successfully registered, introduced, and sustained in a country. When many stakeholders are involved in deciding about the value of a new vaccine, they may all want an economic assessment of the new product performed from their perspective, which may differ from the current conventional analysis of an incremental cost-utility ratio (ICUR) under a specific threshold. This paper highlights some issues arising from having different stakeholders concerned in the vaccine world. It illustrates how an assessment of the potential economic value of a vaccine may need to vary by stakeholder type to address the difference in their concerns. Because under those conditions other evaluation techniques should be used, we propose a tool that facilitates the development and the inventory of a total economic evaluation process for vaccines considering all potential value aspects. We illustrate the application of the tool using rotavirus disease and its vaccination programme as an example, but the approach can be applied to other vaccines as well.

## The many different stakeholders

As the benefits of vaccines extend to a wider population and societal environment beyond the vaccinated individual, many stakeholders are potentially concerned with the value of vaccines [4]:

- *vaccinated individuals* benefit by avoiding disease-specific mortality and morbidity of many different levels of severity;
- *families* or *household units* could benefit from disease prevention in young infants and in elderly people who are especially vulnerable to infection, avoiding both loss of working time and the distress of seeing a relative ill;
- *employers* benefit from avoiding loss of working time due to absenteeism of workers who are sick themselves or have to support a dependent family member;
- *general practitioners* see vaccine-preventable diseases substantially reduced or sometimes disappearing, reducing pressure on consultation times;
- *hospital managers* could benefit from avoiding excessive pressure on staff and ward capacity during infectious epidemic periods;
- *insurers* or *third-party payers* could benefit from a reduction in illness repayments and its consequences;
- the *healthcare system* can benefit by reallocating resources to other priorities than treatment of infectious diseases, and drug resistance may be reduced or avoided by better prevention resulting in a lesser need for treatment; they may be exposed to other challenges such as maintaining the control of infections, developing a strategy of elimination and/or eradication, or most efficient deployment of the vaccination program;
- *central governments* may benefit from reductions in the loss of economic activity due to disease epidemics and reduced social security spending which may benefit employers and employees, and may attract new economic activities such as tourism if they can claim good protection/prevention of infectious diseases;
- *producers or manufacturers* may see appropriate predictability of a sustained vaccine market better for research and development of new vaccines in new indications, making the business attractive and reliable.

**Figure 1.**
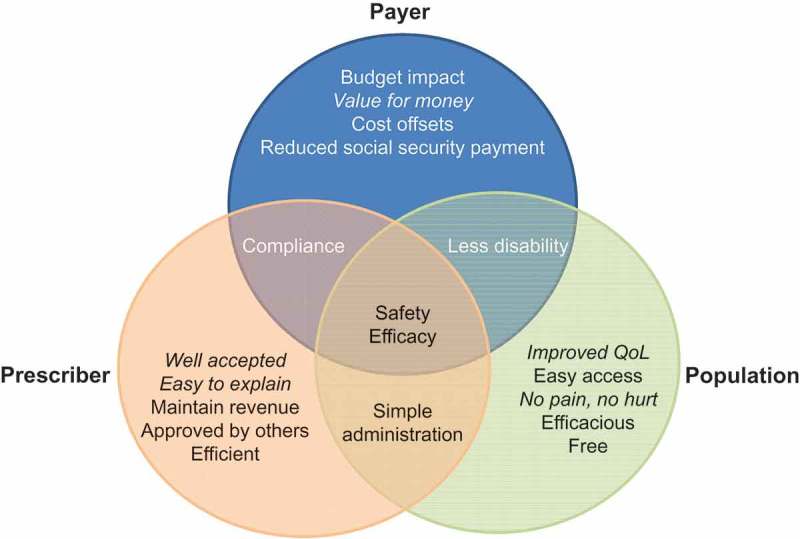
Identifying examples of instrumental and inherent (*italic*) vaccine values by different stakeholder types (payer, population, and prescriber). QoL, quality of life

Figure 1 gives some examples of the instrumental and inherent value of a new vaccine to various stakeholders. The list is not exhaustive but helps to illustrate the variety of value dimensions and the overlap between different stakeholders. The total value could be substantial if all values to all stakeholders are summed up.

In an environment with so many different stakeholders, it is important to understand the views and preferences of each stakeholder type (payer, prescriber, provider, producer, people, and policy) about the value of a new vaccine as they perceive it from their perspective. Each value dimension needs to be substantiated with information appropriate to the specific stakeholder. This is especially critical in an open domain such as public health where everyone can and will have his own perception on the value of vaccines that can be openly and freely communicated through the social media systems that are now widespread [5].

For a new vaccine to achieve high coverage with equal access for all and an appropriate market cost at launch, all the relevant stakeholders have to be simultaneously convinced of its value as each may exercise varying degrees of power or influence. The target group for vaccination is likely to receive the highest direct benefit, and might be expected to recognise the value of the vaccine most readily. However, vaccine recipients do not experience an immediate personal benefit from vaccine administration (in contrast with the symptom relief from a therapeutic intervention). Their assessment of value is likely to depend on their perceptions of the likelihood and severity of the vaccine-prevented disease and their perceptions of the risk of adverse consequences of vaccination, ranging from dislike of injections, to concerns about side effects, health beliefs and cultural/religious issues. These perceptions may be positively or negatively influenced, rightly or not, by other groups in society.

For example, teenage girls and young women are the main group directly affected by human papillomavirus (HPV) vaccination, with direct benefits in the form of protection against HPV infection and cervical cancer. However, other groups such as parents or media commentators may perceive other issues: some mothers of adolescent girls mistakenly value the potential for HPV vaccination to prevent their daughters needing Pap smears, while others may perceive a potential for negative effects on behaviour such as increased promiscuity and increased risk of other sexually transmitted diseases [6,7]. It is important to communicate the value of a new product in a clear and effective manner to all groups with appropriate data for each type of stakeholder. This makes the introduction of a new product such as a vaccine a complex and challenging task [8]. However, evaluation of the total value of the vaccine will enrich its positioning at launch and facilitate pricing decisions that should fairly reflect the total economic value of the new product.

## Total value measurement

To obtain a total value measurement of a new medical intervention against a specific vaccine-preventable disease, one should identify all stakeholders interested in the new intervention and the potential value dimensions (instrumental and inherent) to be assessed for each stakeholder [1]. Ideally the sum of all values should then be compared to all available options, together with the resources spent by option and value type. The option with the highest value in relation to the resources required should be selected, as it would provide the maximum benefit to the target group with the minimum resources needed. One could propose an inverse cost-effectiveness ratio of ranking the options from the highest to the lowest benefit per dollar invested. However, this approach, while attractive, could be an intensive exercise. It might help to elucidate some controversies about the value of vaccines that are under-recognised due to the lack of adequate perception on total value [9,10].

Effective vaccine prevention should normally produce a higher total health gain per individual in a cohort than treatment because it will prevent more disease stages from mild to severe, reduce transmission, and avoid disruption of normal activities. The vaccine might operate over a long timescale, providing protection for years into the future [11]. Vaccination programmes are therefore considered public health initiatives because they actively target an entire at-risk population, rather than passively waiting for patients who already have symptomatic disease to present for treatment [12]. Attempts have been made to develop a clearer inventory of the different types of vaccine benefits (short-term, long-term) that goes beyond the narrow vision of reduction of specific disease burden and consequential improved production (Table 1). However, many additional benefits remain hypothetical and are often difficult to prove over the long-term, such as the benefit of better education and better work resulting from infant vaccination [14]. The longer the timescale until evidence can be measured, the higher the uncertainty grows.

**Table 1.** Total potential value measurements of vaccines [13].

Perspective		Benefit categories	Definition	Individual	Household	Community	Employer	Insurer	MoH	Government
Broad & Narrow		Health care cost savings	Savings of medical expenditures because vaccination prevents illness episodes					x	x	
		Care-related productivity gains	Savings of patients’ and caretaker’s productive time because vaccination avoids the need for care and convalescence	x	x		x			
Broad		Outcome-related productivity gains	Increased productivity because vaccination improves physical and/or mental health	x						
		Behaviour-related productivity gains	Vaccination improves health and survival, and may thereby change individual behaviour, for example by lowering fertility or increasing investment in education	x	x					x
		Health care externalities	Vaccination improves the quality delivery of health care during disease peak periods among those treated for other reasons						x	
		Community health externalities	Improved outcomes in unvaccinated community members, e.g., through herd effects or reduction in the rate at which resistance to antibiotics develops	x	x	x			x	
		Community economic externalities	Higher vaccination rates can affect macroeconomic performance and social and political stability; avoid poverty traps		x	x				x
		Risk reduction gains	Gains in welfare because uncertainty in future outcomes is reduced	x	x	x				
		Health gains	Utilitarian value of reductions in morbidity and mortality above and beyond their instrumental value for productivity and earnings	x	x				x	

MoH, Ministry of Health

The organisational logistics of vaccine prevention differ from those of therapeutic care. Many countries have developed a special healthcare infrastructure for implementing active vaccination programmes for infants and children separate from normal care [15]. Successful vaccine implementation requires a serious shift (mental, organisational, resource use, cost, and infrastructure) from treatment to prevention, which is much more challenging than switching between different treatment-options as commonly occurs in medical practice. So, a vaccine may generate more value benefit than a treatment but it may cost more to implement successfully than drug treatment due to the need to vaccinate as many of the at-risk population as possible to maximise the chances of optimal success.

## Broader perspective

Vaccine positioning in society has evolved over time from the first objective of reducing illness and deaths, to limiting costly medical interventions and outbreaks, to encompass prevention of non-medical disease events (such as infections) overall. This shift in positioning can only be fully understood if the evaluation is conducted at a population and societal level instead of individual gain. Asking a vaccinated subject whether he feels better the day after receiving his vaccine is an anomaly in measuring the vaccine benefit. The individual will never know whether he personally benefited from the vaccine, as he will never know if he would otherwise have experienced an infection that has now been prevented by being vaccinated. It is only by comparing data over time at a group level over which evidence of vaccine benefit can be reported that we can recalculate an average value for individual risk reduction or health gain. Of course, prevention benefits the individual, but the full value can only be demonstrated at the population level. Economic evaluation of vaccines must therefore have the ambition to look preferentially at the next level [16]. To explore the difference between individual and population gain, we consider a broader perspective on how vaccines have been introduced in our societies.

The analysis presented here has focussed on the developed world. Over time, these countries have developed a fully mature healthcare system that currently devotes more resources to treatment than to active medical prevention. The position is quite different when evaluating vaccine prevention programmes in the developing world. Comparing the developed with the developing world helps to define the ultimate goal of introducing new vaccines. Naïvely, it could be said that vaccines are introduced in the developing world to obtain massive health gain in the population by avoiding deaths caused by infectious diseases. In one way that is true, as vaccination will achieve that goal. But this cannot be the final goal of vaccination, as it will be achieved within a short time of implementing the vaccination programme with high coverage and high efficacy, whereas the vaccination programme is recommended to continue year after year, even when there are no more specific deaths to avoid. Therefore, the ultimate goal of vaccination is to control disease recurrence, and to help in the process of disease elimination. So, the objective of vaccines will shift over time, finally reaching its ultimate goal of controlling. That shift in objective is more spectacular in the developing than in the developed world, because the disease burden is so much higher in the former. It is important to retain focus on the final goal, otherwise the vaccination programme may be abandoned. This raises the question of which objective should be used in the economic analysis of vaccines. Should it be addressed with the initial reduction in deaths achieved, or with the disease control phase when disease-specific deaths no longer occur?

**Figure 2.**
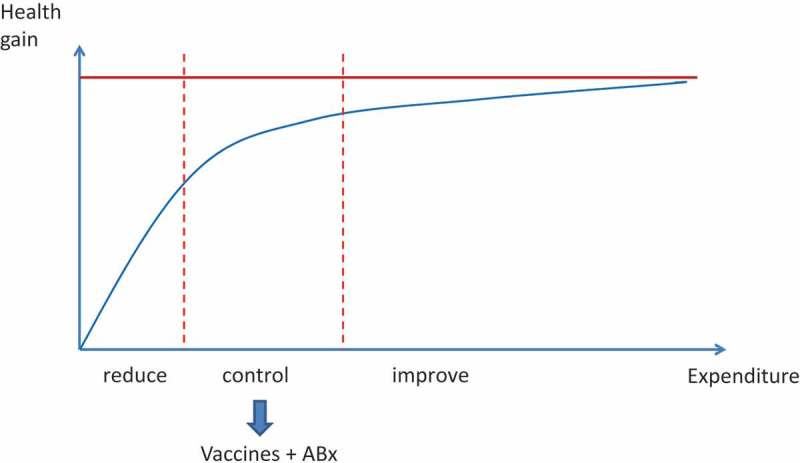
The relationship between health gain and health care expenditures.

**Figure 3.**
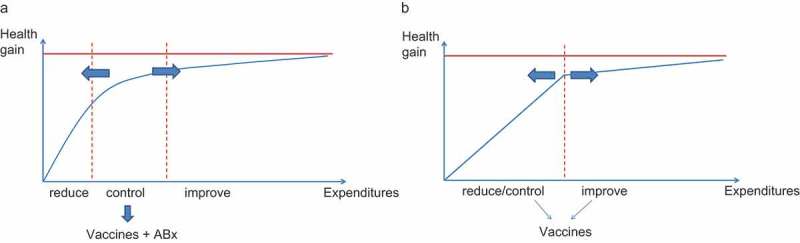
Shifting the use of vaccines from control to reduction with a shift in budget line.

This can be illustrated by schematically plotting the relationship between health gain and incremental budget spent in healthcare (Figures 2 and 3). Figure 2 illustrates what happens if the health problem is large: a small budget can achieve spectacular health gains (left side of Figure 2). For example, this is the situation when education and hygiene measures are first introduced, which are cheap investments but can dramatically improve the health of a population with a high disease burden. An analysis of United Kingdom (UK) mortality data over 250 years illustrates this, as the intervention dramatically reduces the health problem to which people are exposed [17]. The position is different in the next phase, in which the low level of disease achieved by education and hygiene needs to be maintained under continued control. This can be achieved with vaccination and the regulated use of antibiotics (middle part of Figure 2). However, it costs more to achieve a smaller increase in health gain. In mature healthcare markets, the process is no longer about control but competition (right side of Figure 2). The improvements in health obtained by further increases in cost level off and become marginal.

Figure 3 illustrates the progress of introducing vaccines during the past decade in the developing world against diseases such as pneumonia and rotavirus diarrhoea. The environment has shifted from control to a combined effort of control and reduction. Instead of three stages (Figure 3(a)), this shift has created two worlds: one of health gain that will enhance the local economy (the developing world) and one where health gain becomes an area of specific economic interest within the healthcare sector with a high competition between alternatives (the developed world) (Figure 3(b)). For developing countries there is no longer a distinction between reduction and control. By integrating those two elements more money is to be spent to achieve a higher health gain.

The graphs indicate that it is likely that in the developing world the strategy of reduction and control will be very cost-effective because the disease burden is high and the medical infrastructure limited. Any new intervention will result in health gains, so competition plays only a small role. The issue will be in the prioritisation of the healthcare project in the healthcare development programme, making the right choice when the budget is limited. By contrast, in the developed world there will be competition between different healthcare projects offered. Here a common way to proceed is by substitution of projects, and therefore a choice has to be made as to which project creates the highest value for society at an affordable cost. We can summarise this difference between developing and developed worlds by saying that vaccine introduction in the developing world will enhance the health gain for the economy, while in the developed world what will matter is the economic gain that the vaccine will produce in healthcare.

Finally, it should be noted that developing countries are expected to move from reduction following control of the disease to a combined assessment of reduction and control within a period of 15–20 years. This is a much more rapid rate of development than occurred in the developed world, which took more than 150 years to arrive at the present position. The forced time reduction in achieving those health gain goals for the developing world could become an issue if analysis does not correctly assess all the consequences of introducing many vaccines together. For example, it may cause a dramatic baby boom. A holistic approach is therefore warranted to give those societies the chance to develop themselves progressively over time.

## Ways of moving forward

We have previously outlined the reasons why vaccine benefits could be larger when measured at a population and societal level than when assessed as the sum of benefits to vaccinated individuals only [2]. We suggested that current health economic assessment of vaccines using incremental cost-utility analysis (ICUA) based on quality-adjusted life-years (QALY) gains accrued to individuals are unable to provide a full assessment of the different aspects of value offered by the vaccines to many different stakeholders. ICUA is mainly appropriate in an environment where substitution from treatment to prevention is under consideration and where the values of one option are compared with those of the alternative solution. ICUA still has difficulties, because if the new option is accepted following the ICUA criteria, another approved option needs to be displaced to maintain budget equilibrium [18].

In other environments where no substitution can be considered but ‘add-on’ programmes are the message, we may need to think differently. New economic evaluation approaches should be considered in addition to the conventional methods because the focus is not on more value but on priority setting when budgets are limited. New evaluation methods have explored other perspectives, such as considering government instead of the healthcare system as the main decision-maker. This uses return on investment with taxation gain as an outcome measure, expressed as net present value [19,20]. Analyses can also be conducted based on budget constraints with optimisation modelling [21,22]. Cost-benefit analysis (CBA) gives equal monetary weight to all benefits obtained through vaccination [23]. Macro-economic assessments consider avoidance of poverty traps or reduced financial risk at the level of households for diseases such as malaria, tuberculosis, or human immunodeficiency virus (HIV) in developing countries [24].

**Figure 4.**
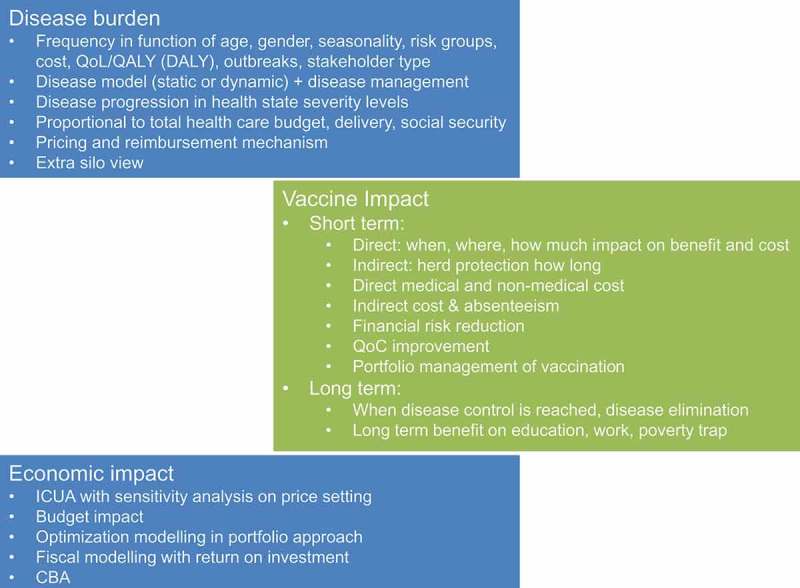
Answering the three critical questions when assessing the full health economic value of a vaccine. QoL, quality of life; QALY, quality-adjusted life-year; DALY, disability-adjusted life-year; ICUA, incremental cost-utility analysis; QoC, quality of care; CBA, cost benefit analysis

Currently, there is no overall consensus on a unified approach to be applied and tested for reporting a total heath economic evaluation of vaccines [25,26]. Different directions are being explored, but there is no developed guidelines that states what to do, when, and how [27]. Yet, decision-makers would like to have solid grounds on which to decide on the next step to take. More than for any other new medical intervention, a satisfactory economic evaluation process for vaccines can only be well developed when basic questions are considered and the answers are incorporated into an overall analysis framework. We propose a more unified approach that does not claim to be the most comprehensive assessment method. It is an instrument that helps to identify potential new value components for the vaccine that requires further exploration. It has been developed based on the three basic questions that need to be answered when assessing its full health economic value (Figure 4).

The first question is to identify the total disease burden under study including all aspects of disease frequency defined by age, gender, seasonality, particular risk groups (e.g., social, work condition, environment, etc.), cost, quality of life, occurrence of outbreaks, and identification of different stakeholders [28]. An accurate model of the natural history of the disease including all information on current disease management approaches by country is an essential tool. The model can be developed using different approaches, including static, dynamic, compartmental or agent-based modelling, mainly depending on availability of data and the primary question to be answered. Disease progression expressed as easily identified health states that differ in cost and/or QALY impact should be reported. Finally, the disease burden should be measured in the context of total healthcare delivery and financing to identify and quantify any extra burden [29]. This information can help to set priorities for the health problems of a country. For example, rotavirus disease should be considered not only in terms of the rotavirus-specific health impact, but also as a health problem in winter when many other infectious diseases appear at the same time in the same age group. What are the consequences of seasonal rotavirus disease episodes in that wider context?

The second question is to understand where and to what extent the new intervention will affect cost and benefit, taking especial care to include medical and non-medical cost items. Non-medical costs may be larger than medical costs if the disease occurs mainly in children, requiring parents to stay at home to care for them. The health benefit could be broader than the impact on the at-risk population alone. For example, direct caregivers may also experience improvements in their quality of life that are often missed in conventional assessments [30,31]. Estimates of the extent and duration of herd protection should be simulated using models. Improvement in quality of care could be considered if enough is known about the disease frequency in relation to other diseases during the same period. The value perception of the new intervention for the stakeholders identified in the previous question should be assessed to define the analysis perspectives. Projects where disease control or disease elimination are considered must be evaluated within a scenario of healthcare development.

The third question is to evaluate the economic impact of the new vaccine compared with the existing situation. The results should be tested under extreme but realistic conditions so that the decision-maker has a better understanding of the strengths and weaknesses of the analysis and the projected impact of the new product in potential real-life settings. The cost range tested here is critical. Cost-effectiveness evaluation can be used, but it should not be the only method. Cost-benefit, cost-consequence, budget impact, and optimisation analysis could offer a more complete economic assessment of the new vaccine, also linked to vaccine portfolio management of infectious diseases in different age-groups [32]. The conditions under which the vaccine would become affordable with various budget impact projections should be tested. Questions related to the ideal uptake scenario of the vaccine and the best place to start should be addressed with the appropriate model design [33].

Answers to the three questions above have provided a framework that has been used for many new vaccines introduced since 2006. They are further discussed in the next sections, using rotavirus disease and its vaccination programme as an example.

## The Cauliflower Value Toolbox

The rotavirus vaccine is a good example to illustrate the importance of collecting relevant new data on disease and vaccine impact in a country to assess its economic value potential. Uptake of this vaccine was generally slow and low in Europe [34]. Decision-makers waited for evidence to appear from the countries that started vaccinating as soon as European marketing authorisation was granted in May 2006, and they expected an important cost reduction for the vaccine because of the marginal benefit demonstrated through modelling exercises [35]. So, evidence of disease reduction was not enough. Other aspects of value for the vaccine had to be explored. This was the main reason for developing a Value Toolbox as an instrument for identifying additional value generated by a vaccine. The tool tries to assemble the critical information needed to construct a full health economic assessment of a new vaccine, structured into one root with three major branches. It has been named the ‘Cauliflower Value Toolbox’ because it resembles a cauliflower branching into many different florets. Each floret provides information about one specific value aspect, and different florets can be combined to measure a specific or a total economic value.

**Figure 5.**
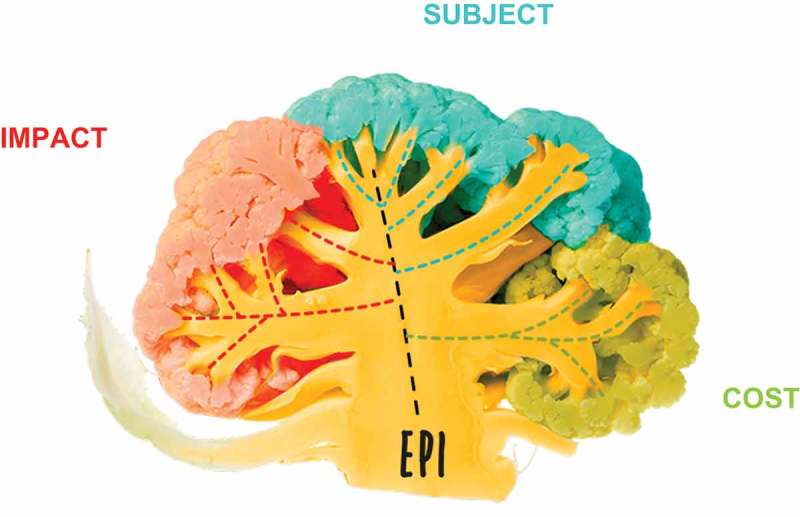
The health economic Cauliflower Value Toolbox. EPI, epidemiology

In its simplest application, the cauliflower tool should be considered as a checklist of the different economic value information (instrumental and inherent) that can be provided for different stakeholders. The various value aspects of the new product are compared with the existing situation, or with the next best current or anticipated alternative. The tool can be considered at global or local level by product. Figure 5 summarises the main domains. Epidemiology information (Epi) forms the root, and the three main branches cover cost information, subject data, and impact data. They are related to the three main questions discussed in the previous section.

**Figure 6.**
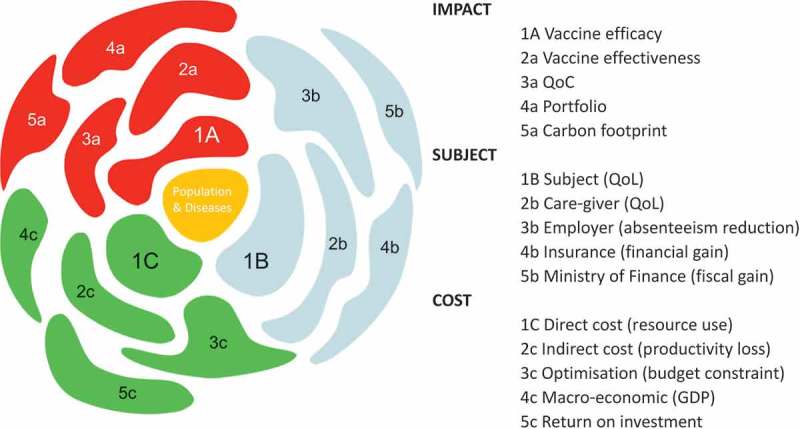
Aspects of value for a new vaccine to be assessed and combined into an economic evaluation. GDP, Gross domestic product; QoC, quality of care; QoL, quality of life

Figure 6 gives an overview of some of the different aspects of outcome information organised by domain (impact, subject, and cost) that can be explored for each vaccine/disease and decision-maker. It also helps to indicate which combination of outcomes could be used for assessing an economic value. For example, the combination of vaccine efficacy (1a), QALY (1b), and direct medical cost results (1c) could be used to construct a basic incremental cost-utility measure helpful for the Ministry of Health (MoH).

Figure 6 is not exhaustive, as it is likely that new florets in each domain could be identified and explored in the near future. The Cauliflower Value Toolbox could be helpful in the future development of a multi-criteria decision analysis (MCDA) programme for vaccines [36,37]. This approach was not initially intended when developing the tool, as the different florets of the cauliflower were developed independently with their own outcome measures, evaluation techniques, and timetables. The tool has the potential to support the development of MCDA, but it needs further exploration.

Table 2 lists the different florets identified so far by domain type, with a definition and an indication of where this outcome information could be used.

**Table 2.** Analysing the health economic Cauliflower Value Toolbox by its essential florets.

Domain	Floret	Definition	Outcome
Population & Disease (yellow in Figure 6)	Central/Root	Epidemiology data by age, gender, at-risk groups & management data.	Basic information for developing a natural disease model with current management impact.
Impact (red in Figure 6)	(1) Vaccine efficacy	Relative reduction in disease/infection incidence rate in the vaccinated group compared with an unvaccinated group.	Obtained through randomised clinical trials.
	(2) Effectiveness (impact) + herd protection	Measured under real-life conditions using case-control techniques or impact studies.	Comparing vaccinated with unvaccinated individuals (historic or not; isolated or not).
	(3) QoC	QoC in hospital care to be measured through bed-day management and people management.	Data should be compared between epidemic periods pre- versus post-vaccination introduction using an impacted summary score.
	(4) Portfolio management	Model-based approach that integrates natural disease history and management among target groups with optimisation analysis by identifying objective functions and specific constraints such as time, budget, logistics for combining different vaccines.	Portfolio management integrates multiple vaccines sequentially over a fixed period of time and budget while achieving maximum health gain.
	(5) Carbon footprint	Total carbon production using vaccination versus no vaccination as an important aspect of societal durability and sustainability of the environment.	Vaccination and reduced vaccine dosing impact carbon footprint in health care compared with no vaccination.
Subject (blue in Figure 6)	(1) Vaccinated subject	QALY to identify how much a disease may impact the utility preference of a subject in different health states.	Allows comparison across different diseases (using a general health-related quality of life instrument with domains common across many disease areas).
	(2) Caregiver	*Healthcare professionals*: overwork, sick leave, absenteeism, QoL. *Informal caregivers*: financial due to absenteeism from work and expenses related to providing informal care (out-of-pocket). QoL impact and well-being level should also be assessed.	*Healthcare professionals*: physicians and nurses may deliver better work and more professional care with fewer errors and better job satisfaction. *Informal caregivers*: family and friends may be affected both financially and in terms of QoL.
	(3) Employer	Reduction in absenteeism on the work floor will benefit the output of the enterprise. May pay less social security contribution for employees.	Number of days being absent from work because of caring for sick family members or because of being sick themselves as an employee.
	(4) Third party payer	Insured people are vaccinated. Their risk for getting the disease is lower and the need for costly medical care is lowered.	Financial benefit because the risk for hospitalisation is reduced by promoting and using vaccination.
	(5) Society	The disease takes away the benefit a healthy population normally provides (healthy workforce, schools, etc.).	Overall benefit considered from different angles/perspectives (direct, indirect, out of pocket, insurance, etc.).
Cost (green in Figure 6)	(1) Direct medical cost	Cost related to resource use for disease treatment (medical visit, laboratory test, medication, hospitalisation, specific intervention). Important to specify the perspective from which the cost is considered (reimbursement, third party, budget holder, patient [out-of-pocket], and society).	An important direct cost driver is hospitalisation to be specified by type (general ward, intensive care, other).
	(2) Non-medical andnon-healthcare cost	Cost that is not medically related to the disease but is a consequence of the disease like loss in income or production loss. Transport costs.	Other expenses than medical cost due to an episode of illness. They might be paid out of pocket over and above their healthcare expenses or lose income due to time off work.
	(3) BIM and BOM	BIM provides budget estimates of the likely impact of the new intervention. Vaccines always impose a high initial investment with cost offset spread over a long period of time. BOM is a modelling exercise that combines different interventions to achieve an objective function or a goal.	BIM identifies the health care budget change over time before and after the introduction of the new intervention. Three scenarios are possible: maintained budget increase after the introduction, budget neutral, savings because of high cost offset. BOM tries to define how to maximise health gain through a combination of new interventions while working under specific constraints, such as fixed budgets.
	(4) Macro-economic	This approach aims at estimating the broad economic consequences (i.e., beyond the sick person or care giver) of illnesses. It is a top-down approach of economic assessment.	Investing in vaccines means investing in human capital which is a foundation for economic growth. The impact of ill health on the economy goes beyond the number of workdays lost. For governments and donors, the choice of investing in health offers an important economic return expressed in GDP improvement.
	(5) Return on investment	This approach aims at estimating the vaccine investment on better tax payments over time because of maintained healthy conditions.	For governments and donors, the choice of investing in health offers an important economic return particularly in taxes.

QoC, quality of care; QoL, quality of life; QALY, quality-adjusted life-year; BIM, budget impact modelling; BOM, budget optimisation modelling; GDP: gross domestic product

## The rotavirus vaccine Cauliflower Value Toolbox, an example

The rotavirus vaccine offers the first example of a concrete application of the Cauliflower Value Toolbox. Before using the tool, the first step is to identify whether the vaccine will be introduced into a well-established healthcare market or into a developing one. The difference is important because different stakeholders dominate each market type. In a well-developed healthcare market the position of the rotavirus vaccine will be influenced by the type of healthcare programme already in place, such as easy access to hospital care and primary healthcare, a high proportion of the parents working, and a social security system in place as well as a private insurance programme [29]. A government may implement the vaccine if they determine there will be an overall picture of benefits at different levels and if there is more than one type of vaccine available. In an environment where the healthcare system still needs to be well-developed (no easy access, no social security system, limited private insurance), the selection of priorities for future development of the healthcare programme needs to be considered based on budget availability [38]. This discussion may happen at a level other than the MoH. The cauliflower tool will then indicate different florets to be explored, compared with an evaluation performed for a well-developed healthcare system. Our focus here is on the value assessment of the vaccine in a developed country such as Belgium, where this vaccine was introduced a few years ago [39].

Tables 3, 4, and 5 illustrate the approach we took using the Cauliflower Value Toolbox. The clinical benefit can also be expressed in monetary terms, with the value for one QALY gained set at €25,000. This will be helpful when we consider the CBA and an additional method of evaluation. Table 3 shows the data used to evaluate the total rotavirus disease burden in a community of 10,000 children aged up to 5 years, including vaccine efficacy with and without herd protection. The evaluation is conducted over 1 year at infection/vaccine steady state (around 5 years after the introduction of the vaccine). In addition (not reported in Table 3) we added a lifetime gain of 85 years life expectancy discounted at 3% per year (=31.6 years) for the disease-specific mortality reduction after vaccination [40]. The investment in quality of care (QoC) improvement without vaccination would require an increment of four additional hospital beds, as estimated from a Belgian study. This leads to a reduced hospitalisation rate for the disease as well as a reduction in specific mortality [41].

**Table 3.** The rotavirus vaccination cauliflower toolbox used in a mature healthcare market: data entry [29,31,40,41].

Cauliflower floret number			% per year	Unit cost (€)	QALY loss/day	VE (%)	Duration (days)
	Cohort	10,000					
1a	VE	Cases	40%			60%	
		Medical visits	15%			75%	
		Hospitalisations	3%			82%	
		Deaths	0.001%			85%	
1b	Subject	Cases	40%		−0.05		6
		Medical visits	15%		−0.10		3
		Hospitalisations	3%		−0.25		4
		Deaths	0.001%		−1.00		365
		QALY		€25,000			
1c	Direct cost	Cases	40%				
		Medical visits	15%	€25			
		Hospitalisations	3%	€2,300			
		Deaths	0.001%				
		Vaccine	86%	€75			
2a	Indirect vaccine effect	Cases	40%			+15%	
		Medical visits	15%			+10%	
		Hospitalisations	3%			+10%	
		Deaths	0.001%			+10%	
2b	Caregiver	Cases	40%		−0.01		6
		Medical visits	15%		−0.05		3
		Hospitalisations	3%		−0.10		4
		Deaths	0.001%		−0.30		365
		QALY		€25,000			
2c	Indirect cost	Cases	40%				
		Working mothers	30%				
		Cost per day lost		€135			4
3a	QoC	Extra investment	+4 beds	€250,000			
		Hospitalisations	2%				
		Deaths	0.0005%				

QALY, quality-adjusted life-year; VE, vaccine efficacy; QoC, quality of care

**Table 4.** The rotavirus vaccination Cauliflower Value Toolbox used in a mature healthcare market, outcome per cauliflower floret added in the analysis.

Cauliflower floret number	Type of analysis	Item	New	Existing	Incremental	ICUA	Cost Vaccine
1a/1b/1c	Simple ICUA	Cost	€862,034	€727,500	€134,534	€25,001	€75.03
		QALY	−3.12	−8.50	5.38		
1a/1b/1c/2a	+ indirect effect	Cost	€799,469	€727,500	€71,969	€11,508	€84.85
		QALY	−2.25	−8.50	6.25		
1a/1b/1c/2a/3a	+ indirect effect + QoC	Cost	€751,445	€747,500	€3,945	€831	€88.37
		QALY	−1.90	−6.65	4.75		
1a/1b/1c/2a/3a/2b	+ indirect effect + QoC + caregiver	Cost	€751,445	€747,500	€3,945	€638	€92.55
		QALY	−2.43	−8.62	6.18		
1a/1b/1c/2a/3a/2b/2c	+ indirect effect + QoC + caregiver+ non-healthcare cost	Cost	€981,485	€1,395,500	−€414,015	Savings	€141.15
		QALY	−2.43	−8.62	6.18		
1a/1c/2b	+ caregiver	Cost	€862,034	€727,500	€134,534	€80,961	€64.22
		QALY	−0.89	−2.55	1.66		
1a/1c/2a/2b	+ caregiver + indirect effect	Cost	€799,469	€727,500	€71,969	€37,693	€72.22
		QALY	−0.64	−2.55	1.91		
1a/1b/1c/3a	+ QoC	Cost	€794,230	€747,500	€46,730	€11,587	€81.32
		QALY	−2.62	−6.65	4.03		
1a/1b/1c/2c	+ non-healthcare cost	Cost	€1,175,666	€1,375,500	−€199,834	Savings	€113.91
		QALY	−3.12	−8.50	5.38		

ICUA, incremental cost-utility analysis; QALY, quality-adjusted life-year; QoC, quality of care

Table 4 presents the results for a range of ICUAs, from a simple combination of the three essential florets (1a/1b/1c) to a more extended societal approach using all the florets indicated in Table 3. With the extended approach, the major driver of the analysis becomes the non-healthcare cost, followed by herd protection and the improvement in QoC. QALY gains appear to be marginal. The last column of Table 4 reports the exercise of estimating the cost of the vaccine at the threshold of €25,000/QALY gained. With all the different attributes included in the analysis, the most extensive analysis will deliver the highest vaccine cost. This analysis is also helpful when performing CBA, as the same cost results should be obtained for the vaccine if all the QALYs are expressed in monetary terms. Table 5 presents the results for CBA.

**Table 5.** CBA for the most extended evaluation of all attributes of the vaccine.

Cauliflower floret refers to Figure 6	CBA	Unit cost/QALY	No intervention	Cost A	Intervention	Cost B	NetBenefit (€)	NetBenefit (%)
	Cohort (10,000)							
	Cases		0.40		0.100			
1b	Subject	€7,500		€82,192		€29,178	€53,014	3%
2b	Caregiver	€1,500		€16,438		€5,836	€10,603	1%
	Medical visit	€25	0.15	€37,500	0.0225	€10,088	€27,413	2%
1b	Subject	€7,500		€30,822		€8,291	€22,531	1%
2b	Caregiver	€3,750		€15,411		€4,146	€11,265	1%
	Hospitalisation	€2,300	0.02	€460,000	0.0016	€96,048	€363,952	23%
1b	Subject	€25,000		€13,699		€2,860	€10,838	1%
2b	Caregiver	€10,000		€5,479		€1,144	€4,335	0%
2c	Non-healthcare cost	€540	0.30	€648,000	0.30	€230,040	€417,960	26%
1c	Vaccine	€141			0.86	€1,213,863	-€1,213,863	−75%
1b	Death (subject)	€790,000	0.000005	€39,500	0.00000025	€7,229	€32,272	2%
2b	Death (caregiver)	€237,000		€11,850		€2,169	€9,681	
3a	QoC	€250,000		€250,000		€0	€250,000	16%
2a	Indirect effect	Yes						
	Total			€1,610,891		€1,610,891	€0	−0.6%

CBA, cost-benefit analysis; QALY, quality-adjusted life-year; QoC, quality of care

The benefit of using the Cauliflower Value Toolbox is that it offers a framework that facilitates the consideration of different benefits and allows different combinations of different florets. This permits a more complete economic value assessment of the vaccine that could be useful for different stakeholders.

In this exercise, we assessed only two different analysis methods, the ICUA and the CBA. Other options of optimisation modelling, portfolio modelling, or fiscal modelling have been explored and reported in the literature [20,21]. We have not included them here because it is difficult to assemble them all into one evaluation programme in one paper.

It is important to notice the difference between ICUA and CBA [23]. Whereas ICUA focusses on QALYs gained and whether the vaccine offers good value for money in improving those specific benefits, CBA highlights the different gains that can be achieved besides quality health gain, such as hospital reduction, a decrease in medical visits and the gain in other costs outside health care. CBA gives equal weight to the different types of benefit, and could disclose more interesting information for a different decision-maker than one preoccupied with health gains. The last column of Table 5 shows the percentage contribution of each item in the analysis. In the overall assessment the main contributors are hospital bed reduction, non-healthcare cost and the QoC-investment. Together they account for 86% of the justification for vaccine cost. The QALY gain contributes less than 14% of the vaccine cost.

**Figure 7.**
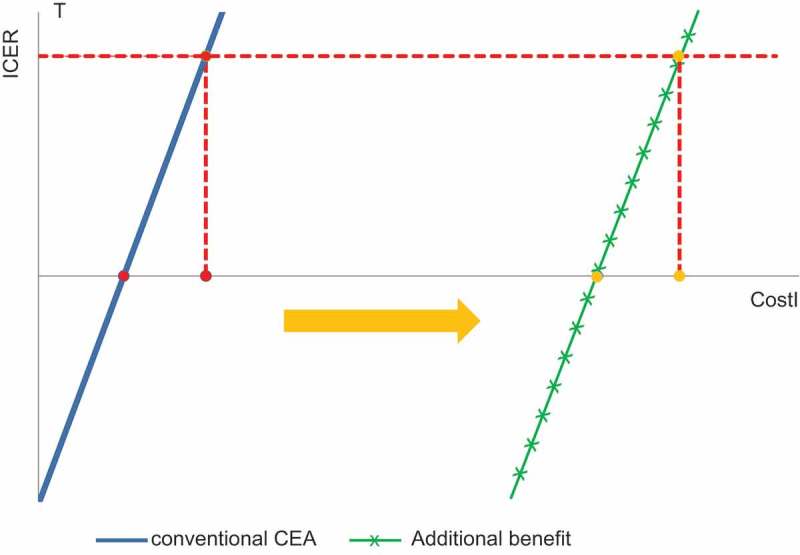
Cost range shift for the vaccine with traditional cost-effectiveness analysis compared with the more extended societal evaluation with the Cauliflower Value Toolbox. ICER, incremental cost-effectiveness ratio; T, threshold; CostI, cost of a new intervention; CEA, cost-effectiveness analysis

Finally, Figure 7 shows the cost range estimated for the vaccine with each type of analysis performed using ICUA. The cost range becomes much higher with the extended approach, because non-healthcare cost, indirect effect, and the QoC gain are all included in the analysis. This has dramatic consequences for the value and budget impact analysis of the vaccine. The cost-neutral point of the vaccine shifts to the right and the budget-neutral point may be reached much earlier than expected with this vaccine if all its value benefits are considered. It may also have consequences for who should pay for the vaccine, as the highest financial benefit could be seen at the level of an employer rather than the healthcare provider.

## Discussion

We argue in this and previous papers [1,2] that vaccines have a broader impact beyond individual benefit when compared with treatment drugs and therefore, their economic assessment should be evaluated at the higher levels of population and society, rather than the individual and patient levels. In addition, budget impact analysis is crucial for vaccines, as an initial high investment is required that will have no immediate pay-off. The pay-off depends on vaccine uptake and coverage, vaccine efficacy, and herd protection. When assessing the economic benefit at a different level than the individual, not one but many stakeholders are interested in understanding the value of vaccines and the decision-making process could be more complex than for treatment drugs. More stakeholders mean more value measures and perhaps more methods of value evaluation to consider. Currently, there is no tool available that integrates all the different aspects presented here (higher level of evaluation, more value types, more stakeholders, more evaluation methods, more decision-makers, and more different environments). Perhaps reflecting the lack of any integration tool, we observe a development of the economic evaluation of vaccines moving in too many different directions without any guidance on the appropriate pathway to follow [27,42]. This is one of the main reasons for developing a supporting framework in which additional vaccine benefits can be investigated more systematically and which ensures that the evaluation process can investigate the many different value aspects offered by a new vaccine.

Applying the cauliflower toolbox to rotavirus disease and rotavirus vaccination yielded some interesting findings. Considering only the conventional approach of ICUA, the QALY benefit and also the cost offset in medical care would be marginal in the developed world. The total value of the vaccine could then be considered poor and that is what decision-makers in European countries have looked at. However, because of the high herd protection level obtained during the first years after vaccine introduction (with a high coverage rate), we observe two benefits that have been poorly investigated and reported in the literature [43,44]. One is the quality of care improvement in the hospital environment during the winter periods after the introduction of the vaccine, which could be critical for hospital managers and healthcare providers as they improve the quality of care of their institutions [45]. The other is the reduction in work absenteeism, which benefits employers and employees, and potentially also the social security system at the governmental level [46]. These additional benefits shift the value of the vaccine to a much higher level that would be difficult to measure using QALYs gained. It also brings the budget impact analysis to a more acceptable range of investment. So, there are reasons to shift our thinking in different directions, and to bring into use a tool that helps to make a more comprehensive inventory of the different components of value in the assessment.

The introduction of this new ‘cauliflower’ approach raises some questions.

First, do we always need to develop the full cauliflower toolbox to assess the total health economic value of each new vaccine, and will the toolbox always be an enrichment as for rotavirus vaccination? This is difficult to answer, as every infectious disease has its particularities and every vaccine has its own benefit criteria. Moreover, it is not always possible to predict the full benefit of a new vaccine through models developed at launch. Accurate prediction of the herd protection effect caused by rotavirus vaccination after the first years of high vaccine coverage could only be achieved using information on nurturing of infants and children in our societies, which was lacking when the vaccine was launched in Europe. The first dynamic models predicted disease elimination after a few years [47]. However, there was no evaluation of different sources of infection in children included in the models, and therefore the models could not assess whether these other sources were governing a baseline rate of rotavirus transmission in the child population, which would have positive and negative consequences. Shifts in healthcare delivery that could occur in environments with bottleneck situations were not assessed in the models at product launch, because we were confined to a narrow perspective of one disease only. The non-healthcare cost benefits for working mothers were not systematically investigated, but were reported in many economic models based on assumptions [48].

The Cauliflower Value Toolbox is an instrument that can help to make an inventory of benefits that can be seen and measured. This can be helpful in environments where there are remaining difficulties in convincing decision-makers about the value of a new vaccine. The instrument can highlight areas that might be missing from the analysis and that might be useful to convince a decision-maker. The cauliflower approach may not be needed for some vaccines because the benefit argument is convincing enough, although it can be helpful to ensure that everything has been considered and evaluated in each potential benefit domain.

Second, is the cauliflower approach still appropriate if the decision is not one of vaccination versus treatment but a choice between different vaccines for the same disease? A full cauliflower assessment is not needed under these circumstances, because vaccine competition is more likely to be a discussion about applying the right cost rather than a full value assessment as compared with treatment [22]. It will be more of an evaluation of details such as the number of doses, the vaccination schedule or the method of vaccine administration, than about different value aspects at different levels. It can be helpful to investigate in greater detail how those detailed differences can be measured and reported, but the cauliflower toolbox is not necessary for this. It is more about direct competition between two interventions in the same environment, which could also be very challenging to conduct.

Third, is the cauliflower toolbox the ultimate way to assess the full benefit of a new vaccine? It may be disappointing that the new tool does not generate one summary number that includes everything, which could be used for ranking different interventions and making an easy selection of the interventions based on that ranking [49]. It would be attractive to do so, but we prefer an evaluation to be more openly debatable. Of course, it is possible to choose a societal perspective that includes everything but there will be many difficulties in the final assessment, such as whether the analysis is fully comprehensive and whether all the data are available. The current difficulties in analysing data with the new regulations in place to obtain access raise a major challenge for this type of full assessment. Through the extended development of informatics systems worldwide that assemble and stock huge datasets about individuals reaching the health care systems we often cannot sufficiently explore the collected information because no consent was given or no contract was in place despite the anonymous reporting of the results. This is the reasoning behind the use of the cauliflower toolbox as the symbol for our approach. Every floret of the cauliflower has its own value and perspective and can be handled separately. There is no need for a single overall value domain. That could be too ambitious, and currently there is no specific demand for it. Instead, by presenting aspects from different perspectives, a decision-maker can better evaluate value in a broader context. An open mind-set with more creativity should allow other value aspects to be discovered.

Fourth, the cauliflower tool lists CBA next to budget optimisation analysis. Should there be a recommendation on when each should be applied, excluding one for the other? The cauliflower toolbox is an inventory of different aspects that can be considered when analysing the economic value of a vaccine. The two evaluation techniques discussed here are applicable for the same vaccine in the same disease, but they are applicable under different circumstances where different questions need to be answered. If the question is well phrased, the application of the tool will identify which method to use. The tool helps to identify that there are different questions to be asked, with different approaches to answer them. It is not an exclusive tool to analyse the problem in one way only. It is possible to use CBA to better understand the cost-value of a new vaccine under the local threshold. Following this an optimisation model with the cost-setting selected from the previous analysis can be applied to identify the health goal that can be reached given the budget constraints. Combination of instruments will facilitate an open discussion on the value of the vaccine, allowing for a more pragmatic approach to understanding potential vaccine benefits.

Understanding the full value of a new vaccine should facilitate its introduction, as it may generate many different benefits across many different aspects of society. That is what the cauliflower is trying to promote. A simple and clearly focussed monitoring programme should be installed once the vaccine has been introduced. This helps to reinforce the results from the cauliflower tool, and to explore new domains where the value of the vaccine can be better assessed.

## Conclusion

Historically, health economic evaluation of vaccines has applied the same framework as developed for comparing therapeutic interventions at the individual level. However, this approach may not be appropriate for vaccines used in public health programmes. Vaccines affect more stakeholders and provide additional benefits that cannot easily be captured in conventional health economic analysis. To help construct a new approach offering a broader view, this paper presents a tool that may clarify areas where potential additional benefits of a new vaccine could be measured, the Health Economic Cauliflower Value Toolbox. It can be applied locally to any new vaccine to identify the main outcome measures driving the economic value, which may differ from the conventional outcomes measured by the QALY gain. These additional gains extend the value of vaccines beyond individual benefits to include wider population benefits. This tool identifies different domains and stakeholders on which the vaccine may have an impact, and expresses the benefit in monetary terms as a simple method of comparison and evaluation.

## Highlights

- The currently adapted method for assessing the economic value of new vaccines (ICUA) may not completely meet the specific requirements for the evaluation of prevention. This may happen because numerous stakeholders are involved in vaccination programs who like to see the analysis performed from their specific perspective.
- We present a new approach to assess the total value and the total cost related to vaccination for the different stakeholders identified: the cauliflower toolbox.
- The cauliflower toolbox helps to map the different stakeholders with their specific value expectations that could be different and broader than the ones for the assessment of therapeutic drugs. The toolbox provides a better understanding and an adequate listing of all the values and costs involved at a population and societal level.
- The new method fully assesses the economic value of vaccines illustrated for rotavirus vaccination. Seven different stakeholders have been identified (the subject, the care-giver, the employer, the hospital manager, the insurer, the health care payer, and the government). Four cost-benefit domains could be assessed including direct medical cost reduction, other cost gain related to the reduction in work absenteeism and tax payment, quality-adjusted life year-gain, and improvement in quality of care. Four evaluation methods could be applied including cost-effectiveness, cost-benefit, optimisation, and fiscal evaluation.

## Note

Based on our study, an oral presentation was presented at the European Society for Paediatric Infectious Diseases (2015) – 33rd Annual Meeting, 12–16 May, Leipzig, Germany; and a poster was presented at ISPOR-EU (2015) International Society for Pharmacoeconomics and Outcomes Research – 18th Annual European Congress, Milan, Italy.
